# Annual severity increment score as a tool for stratifying patients with Niemann-Pick disease type C and for recruitment to clinical trials

**DOI:** 10.1186/s13023-018-0880-9

**Published:** 2018-08-16

**Authors:** Mario Cortina-Borja, Danielle te Vruchte, Eugen Mengel, Yasmin Amraoui, Jackie Imrie, Simon A. Jones, Christine i Dali, Paul Fineran, Thomas Kirkegaard, Heiko Runz, Robin Lachmann, Tatiana Bremova-Ertl, Michael Strupp, Frances M. Platt

**Affiliations:** 1 Population, Policy and Practice Programme, UCL Great Ormond Street Institute of Child Health, London, WC1N 1EH UK; 2 Department of Pharmacology, University of Oxford, Oxford, OX1 3QT UK; 3 Children’s Hospital, University of Mainz Medical Centre, D-55131 Mainz, Germany; 4 NPUK, Vermont House, Concord, Washington, Tyne and Wear, NE13 2SQ UK; 5 Manchester Centre for Genomic Medicine, Saint Mary’s Hospital, Manchester, M13 9WL UK; 6 Orphazyme A/S, Ole Maaløes Vej 3, DK-2200 Copenhagen N, Denmark; 7 Biogen Inc., Cambridge, MA USA; 8 National Hospital for Neurology and Neurosurgery, London, WC1N 3BG UK; 9 Department of Neurology and German Center for Vertigo and Balance Disorders, Ludwig-Maximilians-University Hospital Munich, Campus Großhadern, Marchioninistr. 15, 81377 Munich, Germany

**Keywords:** Niemann-Pick disease type C, NPC, Annual severity increment score, ASIS, Clinical severity scale, Clinical trials, Experimental therapy, Acetyl-DL-leucine, Tanganil

## Abstract

**Background:**

Niemann-Pick disease type C (NPC) is a lysosomal storage disease with a heterogeneous neurodegenerative clinical course. Multiple therapies are in clinical trials and inclusion criteria are currently mainly based on age and neurological signs, not taking into consideration differential individual rates of disease progression.

**Results:**

In this study, we have evaluated a simple metric, denoted annual severity increment score (ASIS), that measures rate of disease progression and could easily be used in clinical practice. We show that ASIS is stable over several years and can be used to stratify patients for clinical trials. It achieves greater homogeneity of the study cohort relative to age-based inclusion and provides an evidence-based approach for establishing inclusion/exclusion criteria. In addition, we show that ASIS has prognostic value and demonstrate that treatment with an experimental therapy - acetyl-DL-leucine - is associated with a reduction in ASIS scores.

**Conclusion:**

ASIS has the potential to be a useful metric for clinical monitoring, trial recruitment, for prognosis and measuring response to therapy.

**Electronic supplementary material:**

The online version of this article (10.1186/s13023-018-0880-9) contains supplementary material, which is available to authorized users.

## Background

Two major challenges when performing clinical trials in rare diseases are low numbers of patients and a high degree of clinical heterogeneity. Therefore, trials risk being statistically underpowered due to differential responses to therapy in a small, heterogeneous group of patients. Current inclusion criteria are predicated frequently on age and clinical severity rather than on comparable rates of disease progression. New approaches to patient stratification for clinical trial recruitment are therefore needed, to improve clinical trial design and aid the interpretation of trial outcomes.

A rare disease with one approved therapy (miglustat) [1–3] and multiple therapies in clinical trials is Niemann-Pick disease type C (NPC) (OMIN 257220). NPC is a lysosomal storage disorder with a current estimated incidence of approximately 1:100,000 live births although it might be more common in adults than previously thought [4]. It is a progressive neurodegenerative disease for which clinical scoring systems have been extensively studied and validated [5, 6]. The substrate reduction therapy drug miglustat is currently the only approved therapy and is available in most countries world-wide, with the exception of the USA. Approval was based on a positive outcome in a non-comparative international clinical trial conducted on 29 NPC patients [3]. Therapies currently or recently in clinical trials include the HSP70 inducer arimoclomol [7]; intrathecal cyclodextrin (VTS-270 2, hydroxypropyl-beta-cyclodextrin) [8], intravenous cyclodextrin (TrapsolCyclo®) and the histone deacetylase inhibitor vorinostat [9]. Inclusion criteria for these trials were differentially based on age and clinical signs ( [3], NCT02612129, NCT02534844, NCT02124083).

We previously conducted a prospective international study validating relative lysosomal volume as a biomarker in circulating B cells from over 100 NPC patients [10]. During the data analysis of the study cohort we identified and characterised six clinical subgroups of NPC patients that differed in their rates of disease progression [10]. The level of disability was assessed in that study using a NPC composite clinical severity score (NPC-CSS) based on multiple neurological subdomains (nine major, eight minor) [5]. Some of the domains are more difficult to evaluate in a comparable fashion between clinical centres due to the requirement for complex examinations (e.g. auditory brain stem response (ABR) that may require anaesthesia in young children). How the total severity score accrues over time reflects the rate of disease progression.

To analyse subgroups of patients with differential rates of disease progression, we developed a metric (denoted annual severity increment score (ASIS)), obtained by dividing the total severity score by the age of the patient providing a measure of the rate of disease progression in individual patients [10]. The resulting value serves as an index of the annual rate of disease progression. We speculated [10] that ASIS could be a potential tool for stratifying patients for clinical trials as it would allow the clinical cohort to be selected such that within a period of the trial (typically 1 year) patients left untreated would have increased their clinical severity score to a measurable and predictable extent, enabling measurement of potential clinical efficacy of drugs in trials. Furthermore, the patients whose disease is particularly severe or extremely mild could be excluded from a trial, using an evidence-based approach, resulting in a more homogeneous sample within the clinical trial cohort. ASIS could also be used to ensure greater equality in assigning patients to different treatment groups in a trial in terms of a pre-stratification. Furthermore, ASIS could facilitate post-hoc analyses to discriminate responders from non-responders, based on the individual progression rates.

A pre-requisite for ASIS to be useful for stratification purposes is that the ASIS score of a given patient remains relatively constant over a prolonged period of time, reflecting a stable rate of disease progression. To test this hypothesis, we have taken advantage of part of the study cohort previously collected [10], but added further longitudinal data points spanning an additional three years. This provided us with a new data set with which we could determine how stable individual rates of progression are over several years and explore the utility of ASIS as a monitoring, recruitment and prognostic tool.

We found that the majority of patients do indeed have stable ASIS scores over several years. In addition, by applying differential ASIS cut-off points, trial cohorts could be stratified to obtain a more homogeneous patient population that, if untreated, would change in clinical score within the typical period of a clinical trial. We have modelled the use of standard inclusion criteria using three real life clinical trials and show that ASIS ensures greater clinical homogeneity. We also present evidence of ASIS’s potential prognostic value for the majority of NPC patients. We further investigated whether individual subdomains or combinations of selected subdomains would correlate with the full neurological composite score. This would address the question of whether fewer tests are adequate for an accurate prediction of the patients’ individual progression. The use of a small number of subdomains has the potential to make the testing less burdensome to clinicians, and to patients and their caregivers. Finally, we applied ASIS to quantify the slowing of the rate of disease progression in NPC patients treated with Acetyl-DL-Leucine, an experimental disease modifying therapy [11].

## Methods

### Clinical study cohort and ethical approval

Thirty-eight patients from two independent international clinical cohorts were studied: 13 from the UK (Manchester) and 25 from Germany (Mainz). They represent a subset of patients from the cohort we previously analysed [10]. In the current study, additional longitudinal data were collected. Research on data obtained from NPC patients were covered by REC/IRB approvals 06/MRE02/85 (UK) and S-032/2012 (Germany). Written informed consent, and if applicable, assent, were obtained in each centre. The NPC-CSS used was the NIH clinical severity scale minus hearing, as hearing was not measured in both centres so was excluded from the analysis [12]. The higher the severity score, the greater the disease burden. The maximum clinical score using this scale for the eight major domains measured (eye movement, ambulation, memory, speech, swallowing, fine motor skills, cognition and seizures [5]) would therefore be 40. Minor domains (cataplexy, behavioural changes, narcolepsy, psychiatric symptoms, hyperreflexia, incontinence, ABR and respiratory signs) were also included, which each had a maximal score of 2. The maximum total severity score a patient could theoretically achieve would therefore be 56 (without hearing). Clinical assessments were conducted by different individuals in the two centres.

### Questionnaire responses

To determine which clinical subdomains related most closely to patient quality of life we conducted two surveys. Firstly, we asked 22 parents caring for a child with NPC (age range of affected child < 1–18 years) and 15 adults with NPC (> 18 years) to answer the following question; “Please rate the following symptoms of NPC in order of impact from 1-9, with 1 having the most impact and 9 the least”. In addition, we asked 16 NPC expert physicians to answer the following question; “Please mark the six domains in the NPC clinical severity scale that in your opinion are of most clinical relevance to the patient”.

### Statistical methods

Chi-squared test was used to compare proportions, Wilcoxon-Mann-Whitney and Kruskal-Wallis tests to compare locations and Ansari-Bradley test to compare dispersions. Correlation was assessed using Spearman’s rank correlation coefficient. Goodness-of-fit was evaluated using the maximum absolute deviation (MAD) from the model’s predictions. Nonparametric kernel density estimators were obtained using a Gaussian kernel with bandwidth selected using Silverman’s rule [13]. We used Friedman’s locally adaptive smoother to represent data-driven joint variations in scatterplots [14]. To assign relative rank order to clinical subdomains questionnaire responses, individual responses were summed and placed in ascending order with 1 being the most important and 9 the least important. All calculations were performed in the R language and environment for statistical computing (http://www.R-project.org) version 3.2.2.

#### *Acetyl-DL-Leucine* treatment of NPC patients

Ten NPC patients (not part of the main study cohort) from Germany and Slovakia (2 females, mean age (standard deviation) 28.1 ± 6.2 years) were assessed immediately prior to and during treatment using eight subdomains of the NPC clinical severity score (Eye Movement, Ambulation, Speech, Swallow, Fine Motor Skills, Cognition, Memory, Seizures).

All patients were on miglustat with the exception of Patient 1. He had a variable filipin staining pattern, clinically typical NPC presentation with vertical supranuclear saccade palsy, cerebellar syndrome, and cognitive impairment. Moreover, he presented with hebephrenic schizophrenia as well as major depression with a suicidal attempt. Just one mutation in exon 6 of the *NPC1* gene, c.709 C > T, p.Pro237Ser has been found. Since both parents have not been found to be heterozygotes, a de novo mutation in *NPC1* gene is suspected. Due to his genotype, miglustat was not covered by the health insurance company hence this patient was not on miglustat therapy unlike the rest of the study cohort.

During the on-treatment phase NPC patients were treated with 3 g/day for the first week and 5 g/day of Acetyl-DL-Leucine (ADLL) (Tanganil, Pierre-Fabre) for the remaining study duration. This was a compassionate use observational study. All study participants and/or guardians of patients gave their informed consent to participation in the compassionate use of ADLL.

## Results

The demographics of the study cohort are summarised in Table 1. Data collected comprised total clinical severity score using the NIH system (see Methods) [12] with 148 measurements in total from 38 patients (minimum repeat measures per patient = 2, maximum repeat measures per patient = 9, median repeat measures per patient = 3) (Table 1). Only patients with complete sub-score data were included in this study. The two centres had similar demographics. There was no statistically significant difference between the age distributions of the two cohorts (*p* = 0.36, Wilcoxon-Mann-Whitney test). The cohort comprised 18 patients with seizures (38% in Manchester, 52% in Mainz, *p* = 0.65, χ^2^ test) and 20 patients without seizures at any point during the study, including patients with a history of seizures, effectively managed with anti-seizure medication (Table 1). Total severity measured using the NIH NPC-CSS [12] at first visit ranged from < 1–33. Patients in Mainz had higher severity scores relative to Manchester with a median score of 9 (Manchester) and 15 (Mainz), *p* = 0.029 (Wilcoxon-Mann-Whitney test). Repeat measurements spanned 0.94–6.36 years with a median of 4 for Manchester and 3.5 years for Mainz (Table 1). Approximately 95% of patients were miglustat treated [3] (Table 1). There was no standardization between centres relating to the stage of disease when miglustat therapy was initiated or treatment duration, although dosing was standardized according to clinical guidelines [15].

**Table 1:** The demographics of the patients in the two clinical centers are summarized

Centre	Total	Median age (years) by centre at first measurement	Female	Male	Median age (years) at first measurement (female)	Median age (years) at first measurement (male)	Repeat measure median	Range of number of repeat measures	Repeat measures in years (median, min, max)	Patients on miglustat therapy at any point in the study	Patients with seizure recorded on at least one visit
Manchester	13	11.16	8	5	12.77	3.92	5	3–9	4.01, 1.05, 6.36	12 (92%)	5 (38.5%)
Mainz	25	12.45	11	14	11.61	16.76	3	2–7	3.50, 0.94, 5.16	24 (96%)	13 (52.0%)

Age in years at first measurement reflects patient age at the first clinical visit included in this study

NPC patients exhibited differential rates of disease progression, as previously defined using latent class mixture modelling [10] to detect subpopulations. Although in common with other LSDs there is a continuum of differential rates of disease progression pragmatically, we have characterised disease progression sub-groups. This facilitates further analyses and fits well with the objectives of randomised controlled trials where it is essential to define inclusion/exclusion criteria.

**Fig. 1:**
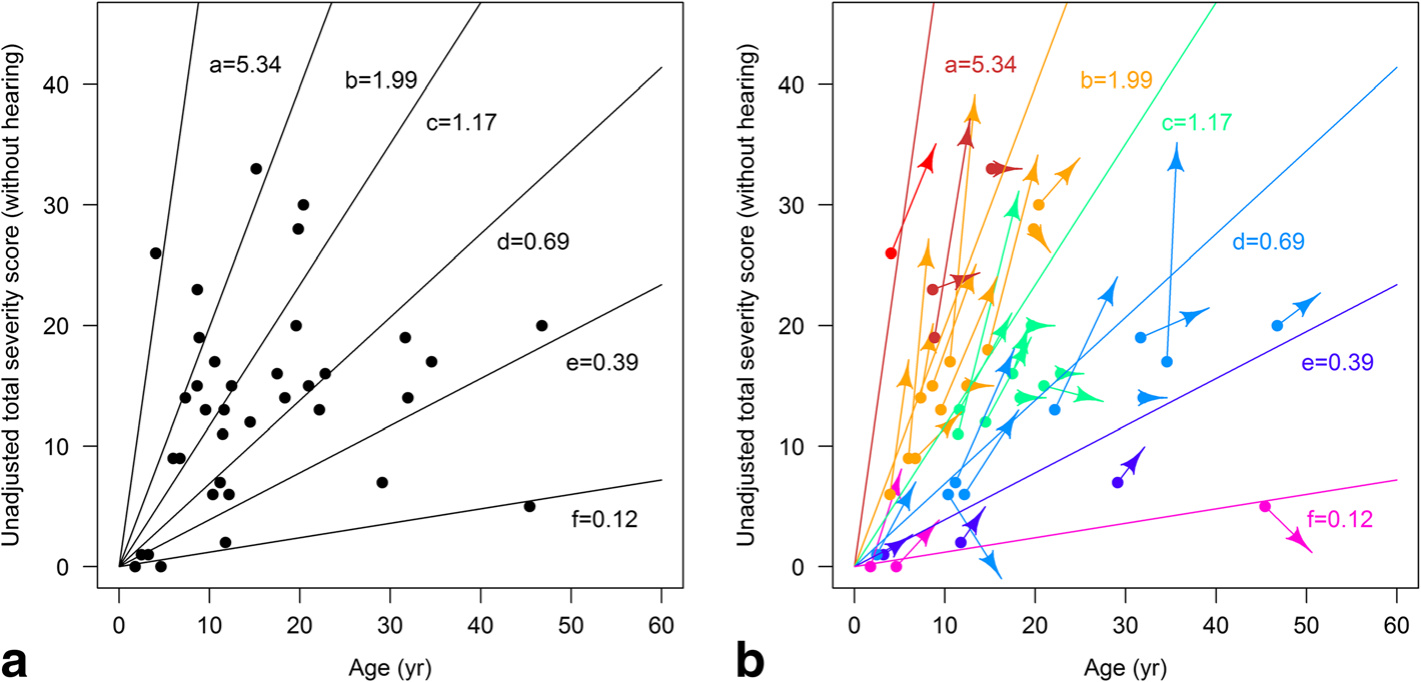
Distribution of the study cohort with each patient represented once (first visit) plotting age against unadjusted clinical severity (minus hearing). **a** Patients fell into six main subgroups defined by lines a-f (latent class mixture regression model). **b** The same data are presented colour coded to lines a-f with arrows showing progression in severity score from first to last visit for each individual in the study

We therefore repeated latent class mixture modelling on this study cohort by plotting patient age against total severity score (minus hearing) using the NIH clinical severity scale [12] (Fig. 1). Each patient appears only once, which was arbitrarily defined as his or her age and severity score at first visit (Fig. 1a). As defined previously [10], patients were clustered into six subgroups based on differential rates of disease progression (Fig. 1a lines a-f). The numbers (Fig. 1a) are determined by fitting a latent class mixture regression model, as detailed in [10]. Arrows show severity progression for each patient from first to last visit (Fig. 1b). The coloured lines and arrows represent the different subpopulations (**a-f**) determined in Fig. 1a to which patients were assigned. This classification allocated each patient to the subgroup (a-f) to which their initial severity score was closest. Most patients’ trajectories were well determined by their initial value and most trajectories followed one of the six subgroups defined in our previous analysis [10] (Fig. 1b). Most of those that deviated moved into the territory of the next adjacent trajectory. Only one patients changed classification category by more than one category.

**Fig. 2:**
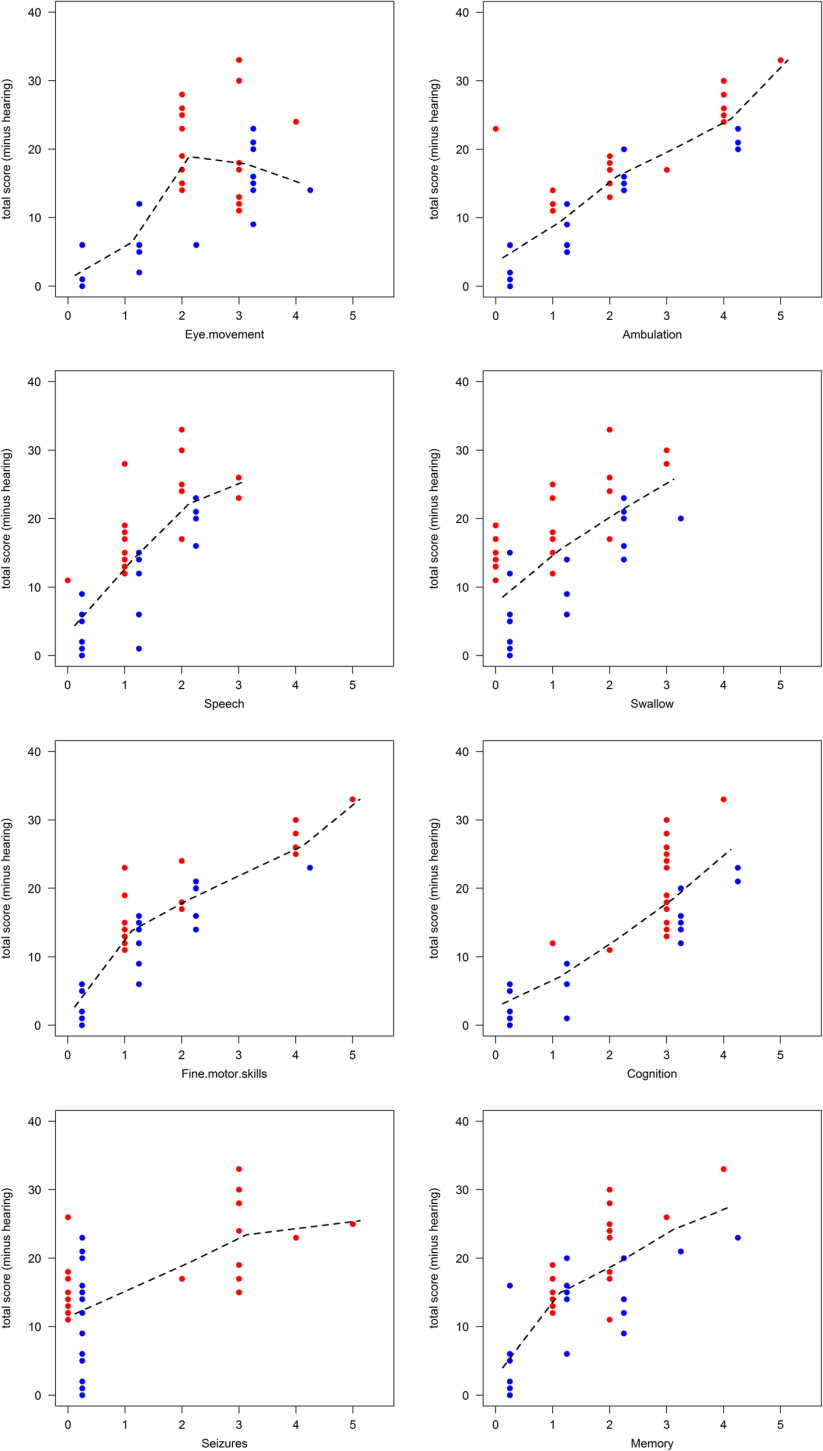
Each of the main clinical subdomains (x-axis) were plotted against total severity score and the coefficient of variation (Spearman’s correlation coefficient) determined (see Additional file 1: Table S1). The dotted line represents the scatterplot’s Friedman’s adaptive smoother. Each patient appears once. Red dots are patients with seizures

The total severity score is the sum of the neurological subdomain scores [12] and can be time consuming and technically challenging to ascertain in standard clinical settings. We therefore investigated whether one or more select subdomains could substitute for total severity score (comprising 8 major and 8 minor subdomains) by plotting total severity score against individual subdomain scores and calculating the Spearman’s correlation coefficient (Fig. 2 (dotted lines corresponding smoothers), Table 2, Additional file 1: Table S1, Additional file 2: Table S2 and Additional file 3: Table S3). There was a significant correlation (*p* < 0.001) between total severity scores and single subdomains ranging from 53 to 89% concordance, with the exception of more binary clinical signs such as e.g. seizures (60%) (Fig. 2 and Additional file 1: Table S1). We then compared the correlation when we combined two subdomains (*m* = 2) with total severity scores. Correlations rose to range from 71 to 93% (Additional file 2: Table S2). Combining any three subdomains (*m* = 3) improved the correlation further (Additional file 3: Table S3) with correlations ranging from 84 to 96%. As the subdomains we were comparing were contributing to the total severity score we also calculated these correlations excluding the subdomains we were analysing from the total severity score. This is summarised in the right-hand column in Additional file 1: Table S1, Additional file 2: Table S2 and Additional file 3: Table S3. Removing the subdomains from the total scores did not greatly affect the correlation coefficient in most cases. All correlations coefficients shown in Additional file 1: Table S1, Additional file 2: Table S2 and Additional file 3: Table S3 were highly significant (*p* < 0.0001).

**Table 2:** Spearman’s pairwise correlations between major subdomains of the Niemann-Pick type C clinical score

	Eye movement	Ambulation	Speech	Swallow	Fine motor skills	Cognition	Seizures	Memory
Eye movement	1.000	0.600	0.472	0.598	0.606	0.600	0.051	0.509
Ambulation	0.600	1.000	0.679	0.658	0.891	0.810	0.412	0.704
Speech	0.472	0.679	1.000	0.689	0.727	0.728	0.331	0.551
Swallow	0.598	0.658	0.689	1.000	0.754	0.561	0.230	0.485
Fine motor skills	0.606	0.891	0.727	0.754	1.000	0.786	0.430	0.754
Cognition	0.600	0.810	0.728	0.561	0.786	1.000	0.354	0.724
Seizures	0.051	0.412	0.331	0.230	0.430	0.354	1.000	0.366
Memory	0.509	0.704	0.551	0.485	0.754	0.724	0.366	1.000

In order to understand which of the subdomains relate most closely to quality of life, sixteen NPC expert clinicians and thirty-seven members of patient organisations (typically parents of affected children and affected adults) were asked how they valued changes in the major clinical subdomains, using a simple questionnaire (see Methods). The data are summarised in Table 3. The NPC clinical experts selected (in rank order starting with the most important) the following subdomains; ambulation, swallowing, speech, cognition and fine motor skills. The patient organisation members selected ambulation, speech, swallowing, fine motor skills and cognition as the most important subdomains from their perspective as carers (starting with the most important). The expert physicians and the parents showed 100% agreement on the five most important subdomains. The only difference was the relative rank order of speech and swallowing, with physicians giving more weight to swallowing than parents/patients.

**Table 3:**
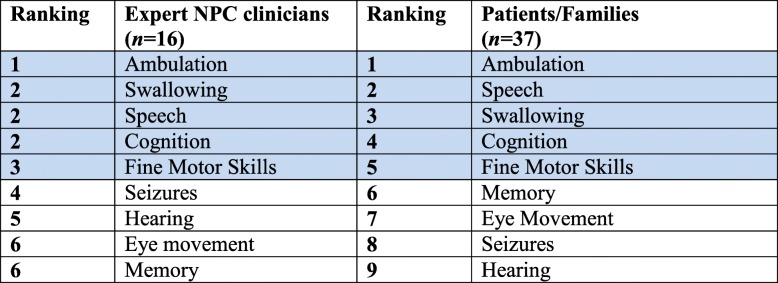
Ranking of clinical subdomains most important to parents of NPC1 patients and expert clinicians. The top 5 subdomains selected by each group completing the survey are shaded in blue

Neither group weighted seizures as highly important. The severity of seizures can be confounded by anti-epileptic medication, which makes it problematic for use as a representative subdomain. We therefore compared how well the five components selected by clinicians and parents/patients correlated with total severity. This was calculated with and without inclusion of the components in question in the calculation of the total score. Spearman’s correlation coefficients were 93% and 71% respectively.

**Fig. 3:**
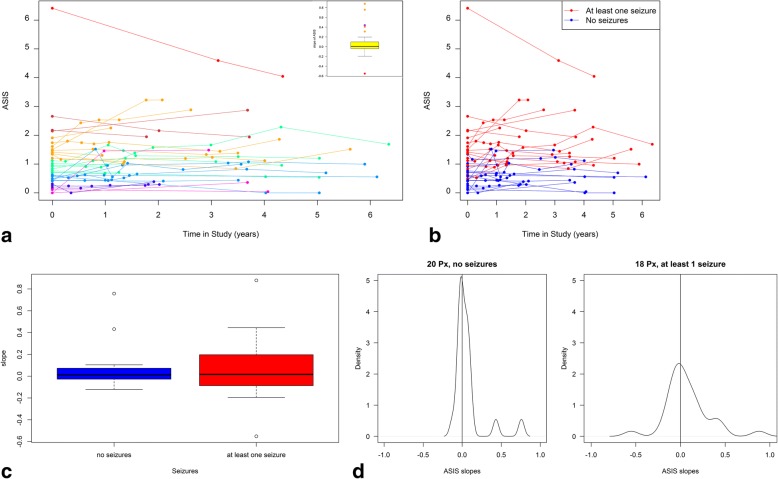
**a** ASIS scores (total severity/age for every time point) were plotted for each patient over time (years), colour coded to reflect the subgroups from Fig. 1a**. b** The same data were plotted colour coding patients with seizures in red. **c** Box and whisker plots demonstrating the greater variability of ASIS scores in the seizure versus non-seizure group. **d** Density plot of ASIS score variation around zero for seizure, non-seizure groups

The ASIS score (total severity score/age) was then calculated for all 38 patients at each clinical assessment visit (Fig. 3a). We found that the majority (82%) of patients had stable ASIS scores over several years. Stability was defined by inclusion within the whiskers of the box plot insert (Fig. 3a). The median ASIS score for the cohort at first visit was 0.96 (Interquartile range (IQR) = (0.49, 1.52)). The slopes of individual ASIS scores (box and whisker insert plot) were very close to zero, consistent with a stable rate of disease progression over the time period measured (median, 0.004, (IQR = (− 0.0038, 0.094)) (Fig. 3a, inset). Six outliers had positive rate of change with one outlier exhibiting a decrease in rate of change. Interestingly, patients with seizures were over represented in the non-stable ASIS group (71%) compared to the total cohort (47%). The patient with the highest score showed a downward slope, reflecting the fact that they had responded to seizure medication and their severity score/ASIS had declined accordingly.

The patients were then stratified based on whether they had seizures and their ASIS scores plotted against time. Patients with lower ASIS values (slower rate of disease progression) tended to be the patient group not presenting with seizures (Fig. 3b). The median slope for the non-seizure group was − 0.003, (IQR = (− 0.03, 0.07)) and for the seizure group (defined as at least one seizure during the study period) was 0.02, (IQR, − 0.08, 0.19) (Fig. 3c). The Wilcoxon-Mann-Whitney test comparing medians was not significantly different (*p* = 0.74) and the Ansari-Bradley test for dispersion (spread of data) was not significant (*p* = 0.09) when comparing the slopes of ASIS for the seizure/non-seizure groups (Fig. 3d).

**Fig. 4:**
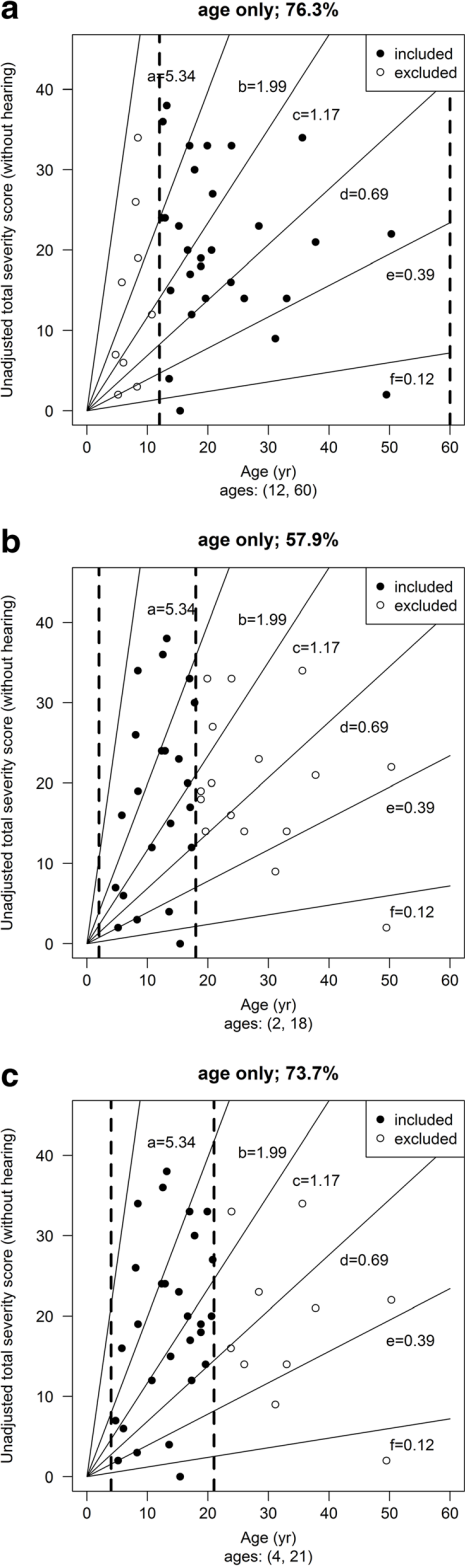
The inclusion criteria for three clinical trials were modeled on plots of age versus total severity score (minus hearing). **a** Miglustat trial, ages > 12; **b** Arimoclomol trial ages 2–18 and **c** 2-Hydroxy-beta-cyclodextrin ages 4–21. The plots depict the study cohort with these different inclusion criteria indicated with the dotted lines. Included patients are in black, excluded patients in white

To test the potential utility of ASIS for patient recruitment to clinical trials we modelled patient selection for a hypothetical clinical trial based on our study cohort. We compared how ASIS scores compared with conventional age/severity score inclusion criteria based on three real life clinical trials (Fig. 4). The inclusion criteria for the three trials modelled in this study are those used in the pivotal miglustat clinical trial (patients older than 12 years [3]) and the current trials for Arimoclomol (age 2–18) (https://clinicaltrials.gov/ct2/show/NCT02612129) and intra-thecal 2-hydroxypropyl-β-cyclodextrin (age 4 to 21) (https://clinicaltrials.gov/ct2/show/NCT02534844) (Table 4). All three-age inclusion criteria modelled onto the study cohort included patients from all severity groups and included 76.3% (12–60 years inclusion), 57.9% (2–18 years inclusion) and 73.7% (4–21 years inclusion) of the study cohort respectively (Fig. 4a-c).

**Table 4:** Summary of age inclusion criteria and requirements for neurological signs. The neurological sign requirements are unique to each clinical study

Trial	Selected Inclusion Criteria		References/Clinical Trial Identifier
	Age	Neurological sign	
Miglustat	> 12	+	[3]
Arimoclomol	2–18	+	NCT02612129
2-hydroxypropyl-β-cyclodextrin	2–25	+	NCT02534844

**Fig. 5:**
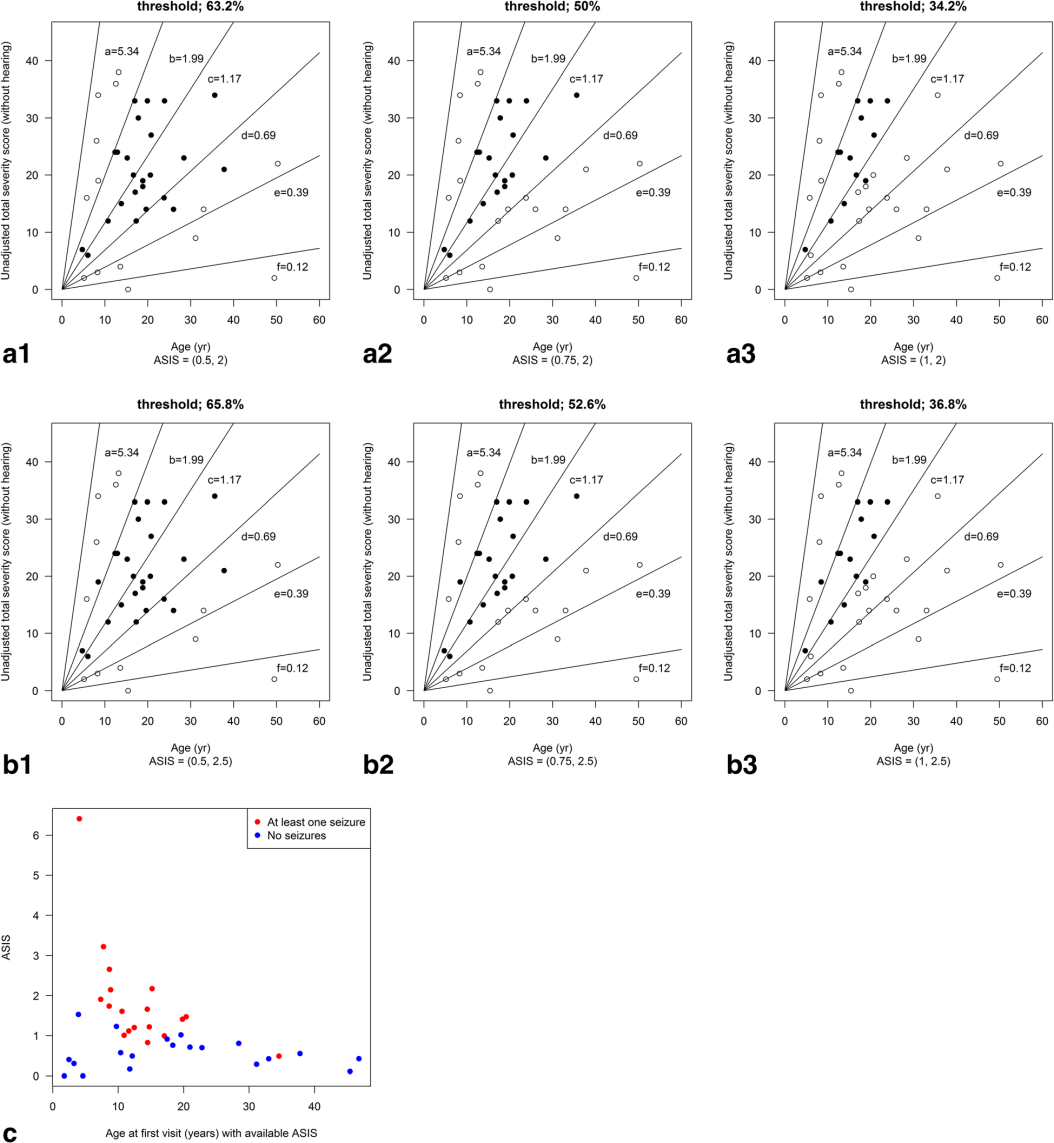
Inclusion criteria based on ASIS scores are plotted using three thresholds (ASIS 0.5 to 2; 0.75 to 2; 1 to 2) plotted in **a1-a2** and **a3** respectively. The ASIS bands plotted were extended to include 0.5–2.5, 0.75–2.5 and 1–2.5 (**b1, b2** and **b3** respectively). **c** The ASIS thresholds were plotted to demonstrate their influence on seizure/non-seizure cases (non-seizure cases in blue, seizure cases in red)

We then modelled patient inclusion solely based on ASIS. Three different ASIS inclusion criteria thresholds were applied focusing on ranges that excluded very mild and very severe patients (0.5 to 2; 0.75 to 2; 1 to 2) (Fig. 5a1-a3). As predicted, all three ASIS criteria resulted in the inclusion of patients in the middle of the clinical severity range, eliminating the mild and severe extremes of the cohort. The percentage of eligible patients that ASIS defined in our cohort was 63.2%, 50.0% and 34.2% for ASIS bands of 0.5–2, 0.75–2 or 1–2 respectively. The ASIS score range of 0.5–2.0 (63.2%) encompassed the broadest range of patients in severity groups b-d and resulted in the largest group of included patients (Table 5). The pairwise comparisons of age-defined and ASIS-defined inclusion are summarised in Table 5, presented as percentage of patients excluded and included in between-group comparisons. For example, if we compare age inclusion of 4–21 with ASIS inclusion of 0.5–2, 47.4% of patients would be recruited to both trials and 10.5% excluded from both trials. We also modelled ASIS bands with an extended upper limit from 0.5–2.5, 0.75–2.5 and 1–2.5 (Fig. 5b1-b3). This expanded the percentage of eligible patients slightly to 65.8%, 52.6% and 36.8% respectively. The inclusion of patients with and without seizures is presented in Fig. 5c. ASIS bands with lower thresholds encompassed a greater percentage of non-seizure cases, consistent with the fact that the non-seizure group tended to have lower ASIS scores (Fig. 5c and also Fig. 3b). The highest proportion of patients meeting the inclusion criteria was for ASIS scores ranging from 0.5–2.5 (65.8%).

**Table 5:** Summary of patient’s eligibility to be included (% included) based on different ASIS thresholds compared with three clinical trials based on age inclusion (upper panel). The lower panel summarise between group comparisons showing the percentage of patients included and excluded when comparing ASIS inclusion criteria versus age inclusion criteria

	Inclusion criterion	% Included	
ASIS-defined	0.5–2	63.2	
	0.75–2	50.0	
	1–2	34.2	
	0.5–2.5	65.8	
	0.75–2.5	52.6	
	1–2.5	36.8	
Age-defined			
Miglustat trial criteria [3] *n* = 39	> 12	76.3	
Vtesse/Sucampo trial criteria *n* = 44	4–21	73.7	
Orphazyme trial criteria *n* = 39	2–18	57.9	
	Between groups comparison (% Patients excluded, included)		
	Age-defined		
ASIS-defined	> 12	4–21	2–18
0.5–2	15.8, 55.26	10.5, 47.4	10.5, 31.6
0.75–2	15.8, 42.1	18.4, 42.1	21.1, 28.9
1–2	18.4, 28.9	23.7, 31.6	31.6, 23.7
0.5–2.5	13.2, 55.3	10.5, 50.0	10.5, 34.2
0.75–2.5	13.2, 42.1	18.4, 44.7	21.1, 31.6
1–2.5	15.8, 28.9	23.7, 34.21	31.6, 26.3

**Fig. 6:**
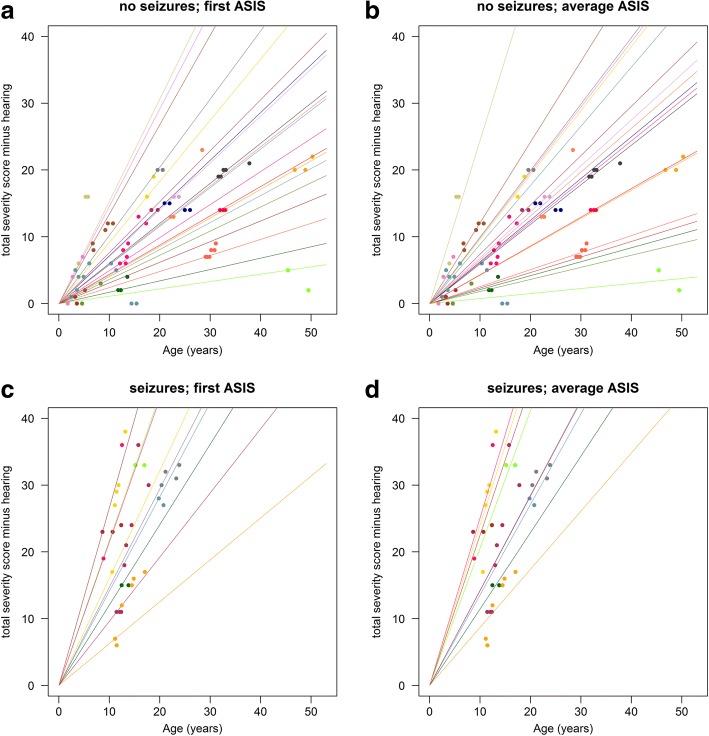
Plots of total severity score minus hearing defined from either a single ASIS score (standardised to the first data point for each patient) or using a mean ASIS score (repeat measures). The four scatterplots represent patients with and without seizures, and with predicted trajectories calculated based on a single ASIS determination at first visit (**a**) (without seizures) and (**c**) (with seizures)) or where predicted trajectories are based on the average of all available ASIS scores for each patient (**b** and **d**) for patients with and without seizures, respectively. Each patient has been assigned an individual colour and each visit is represented by a data point (multiple points per patient)

**Fig. 7:**
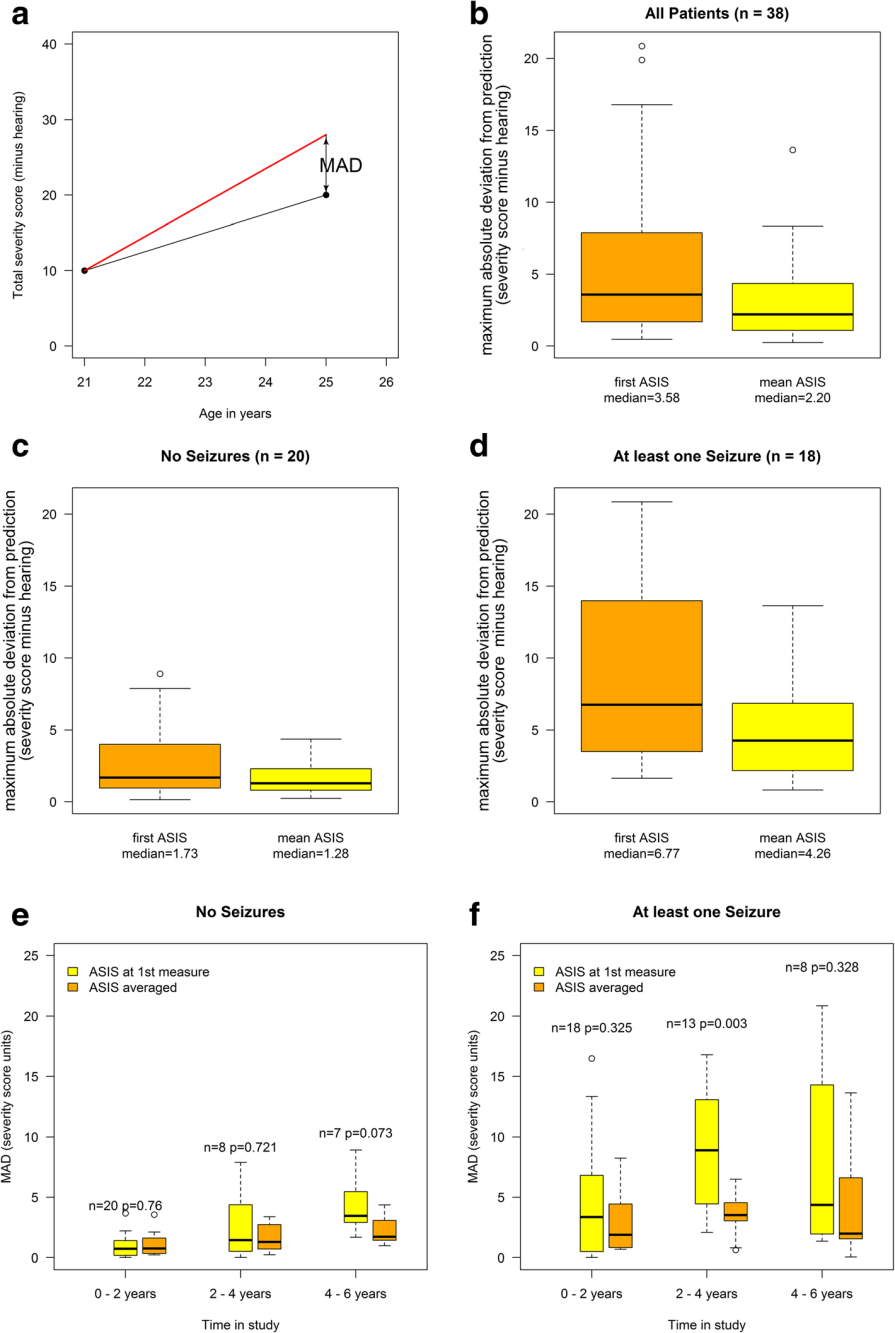
The mean of the maximum absolute deviations (MAD) was calculated for each individual in relation to the predicted trajectory, based on a single or average ASIS score relative to actual severity (panel (**a**) illustrates MAD in a single case). The black arrow shows the point of maximum deviation. The goodness-of-fit criterion, based on MAD, is depicted in the box and whisker plots for all patients (**b**) and patients with no seizures (**c**) and patients with seizures panel (**d**)). The effects of time on MAD determination depicted as box and whisker plots for patients without seizures (**e**) and with seizures (**f**). The analysis was performed for repeat measures spanning data over 0–2 years, 2–4 years and 4–6 years. Each patient only contributes once

In addition to aiding selection of patients for clinical trials, the other potential application of ASIS is as a prognostic tool. We therefore modelled predicted (based on ASIS) versus actual (measured) clinical severity score over time. We compared trajectories of total severity score, minus hearing, defined from either a single ASIS score (standardised to the first data point for each patient) or using a mean ASIS score, taking advantage of repeat measures to see which method was most robust. We generated four scatterplots representing patients with and without seizures, and with predicted trajectories calculated based on a single ASIS determination at first visit (Fig. 6a (without seizures) and Fig. 6c (with seizures)) or where predicted trajectories are based on the average of all available ASIS scores for each patient (Fig. 6b and d for patients with and without seizures, respectively**)**. We then fitted individual trajectories defined as straight lines without intercept and with their slope determined by either the first value or the mean of all the available ASIS values. The graph shows the measured points for each individual over time colour coded to match their predicted trajectories based on ASIS (single or average). The mean of the maximum absolute deviations (MAD) was calculated per individual in relation to the predicted trajectory, based on a single or average ASIS score relative to actual severity (a single example is shown in Fig. 7a). The goodness-of-fit criterion (seizure and non-seizure groups combined) based on MAD indicated that the average ASIS, over all available measurements, had better predictive power (median MAD = 2.20, IQR = (1.10, 4.27) than a single ASIS ascertainment (median MAD = 3.58, IQR = (1.68, 7.62)) (difference between mean and single ASIS *p* = 0.027 (Wilcoxon Mann-Whitney), difference in dispersions (Ansari-Bradley) between mean and single was non-significant *p* = 0.642) (Fig. 7b). When analysing the non-seizure group based on average ASIS measurements the value came very close to 1 severity unit (1.28, IQR = (0.82, 2.27)) (neither location nor dispersion were significantly different in this group) (Fig. 7c, and Table 6). Using instead the first available ASIS score the median of this prediction was higher at 1.73 (IQR = (1.09, 3.90)) (*p* = 0.086) (Fig. 7c). The seizure group data were more variable with median ASIS scores giving a higher deviation than the non-seizure group in predicted and actual data, using the average ASIS scores (median MAD = 4.26, IQR = (2.38, 6.77) (significantly different location *p* = 0.037, but not dispersion *p* = 0.397) and also when using the first available ASIS score (median MAD = 6.77, IQR = (3.59, 13.96)) (*p* = 0.037) (Fig. 7d and Table 6). The repeat measures spanned different periods of time for individual patients. This allowed us to investigate how well ASIS predicted future clinical severity of patients over three time periods (up to 2 years, 2–4 years and 4–6 years) (Fig. 7e and f). Firstly, we investigated how MAD values differed between predicted and actual scores for patients without seizures using first or mean ASIS scores (Fig. 7e). The ASIS scores (when combined and the mean determined) were not significantly different over six years (0–2, 2–4 and 4–6 years) relative to single ASIS determinations (*p* > 0.073), whereas single ASIS based predictions were more variable when the same analysis was performed on patients with seizures (Fig. 7f). The data were more variable based on single ASIS predictions but mean ASIS scores also gave comparable predictions over the different time intervals, although at 2–4 years the single and mean MAD values were significantly different (*p* = 0.003). Taken together these data suggest that ASIS is a reliable predictor of progression of clinical severity over a six-year period, particularly if averaged ASIS scores are used as the basis for the prediction, and that it is more robust in the non-seizure group i.e. before seizure onset when there is less phenotypic variability.

**Table 6:** Summary of MAD scores (in clinical severity units) comparing predicted and actual disease progression based on first or mean ASIS scores for seizure and non-seizure groups

Patient Group	First ASIS (severity score units)	Mean ASIS (severity score units)
No seizures	1.73	1.28
At least one seizure	6.77	4.26

**Fig. 8:**
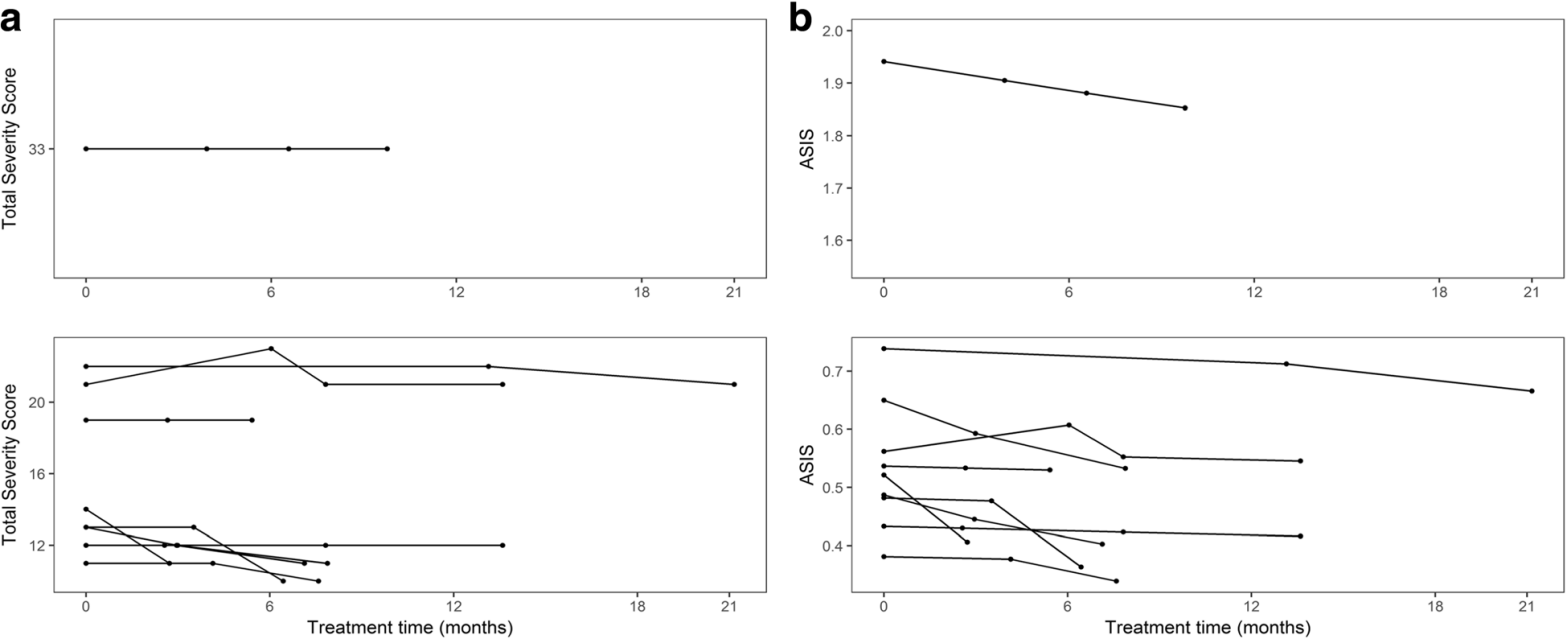
**a** Total Clinical Severity Score and **b** ASIS scores (total severity score/age at time of assessment) of NPC patients post-commencement of treatment with 5 g/day Acetyl-DL-Leucine. The initial severity score (and hence ASIS) of one patient was notably higher than that of the other nine. This patient’s data is therefore provided as a separate graph. Each assessment is represented by one data point

ASIS scores were also used to quantify the effect of an experimental therapy for NPC disease progression using the modified amino acid Acetyl-DL-Leucine (ADLL). ADLL has been used (as Tanganil®) in France and selected former colonies for around 60 years for the treatment of acute vertigo. A recent publication demonstrated that short-term (4 weeks) treatment with 5 g/day ADLL was associated with symptomatic improvement (most notably in ataxia) in 12 NPC patients [11]. Additionally, the parents/carers of NPC patients anecdotally reported cognitive improvement on-treatment, suggesting that ADLL may have clinical benefit beyond amelioration of symptoms of ataxia [11]. The effect of longer-term ADLL treatment on disease progression in NPC patients was therefore assessed (Fig. 8a and b, total severity scores and ASIS on treatment respectively). Ten NPC patients were treated with ADLL (5 g/day). The median ages at first visit were 27.3 years (minimum 17, maximum 37.4) for males and 27.7 years (minimum 20, maximum 35.4) for females. At baseline the median ASIS value was 0.53 (minimum 0.38, maximum 1.94, mean 0.67). Patients were treated with ADLL for a median length of 7.7 months (maximum 21.16, minimum 2.7 months). All patients were assessed at least once during the ‘on-treatment’ period. Six patients were assessed three times on-treatment, and a further three were assessed four times on-treatment and one patient was assessed twice. ASIS at final measurement ranged from 0.3393 to 1.8526 (median 0.6054, mean 0.473) (Fig. 8). A linear mixed-effects model fitted with patient identification as an intercept random effect showed a significant improvement (reduction) in ASIS on-treatment (*p* < 0.0001) (Fig. 8b). On average, ASIS was reduced by an average of 10.4% per year (average rate of change of − 0.06 units per year), consistent with ADLL slowing the rate of disease progression in NPC patients. As this was an open-label study a placebo-controlled trial will be required to determine efficacy in the future and measurement of ASIS has the potential to be a useful metric to evaluate in future clinical trials.

## Discussion

In rare diseases the combination of low patient numbers and high degree of clinical heterogeneity makes diagnosis, prognosis, clinical monitoring and recruitment to trials challenging. In this study, we have explored the potential utility of a clinical metric in the rare neurodegenerative lysosomal storage disease, NPC. As NPC currently has one approved drug (miglustat) and multiple trials in progress we have focused on this disease to test and model new methods of patient stratification.

Currently, most clinical monitoring uses non-weighted severity scales based on multiple neurological subdomains contributing to a total composite severity score. Conducting these assessments is time-consuming and technically demanding. As a result, the full composite clinical score is not routinely ascertained for all patients in standard clinical settings. Patients with a given clinical severity score may have radically different ages and different rates of disease progression (Fig. 1). As a result, a trial based primarily on age inclusion criteria will include patients spanning the disease spectrum, resulting in a heterogeneous group that includes the two clinical extremes. This, in combination with low patient numbers, poses a significant challenge in proving whether new therapies are disease modifying or not. This is because the trial cohort includes patients so mild their clinical score would not change within the trial period and those with advanced disease that are less likely to respond to therapy due to a high burden of pre-existing irreversible pathology.

Clinical trial recruitment to date in NPC has been based on age inclusion criteria, coupled with neurological signs (reflected by a clinical severity score above zero), to eliminate the pre-symptomatic patients, but does not take into account differential rates of disease progression or achieve any further stratification.

Therefore, to test a new stratification method we have assembled an international NPC study cohort, based on patients from two clinical centres in Europe.

We began by investigating whether rate of disease progression is a more useful metric for stratification for clinical trials rather than age. In our previous study [10] we developed a score we termed ASIS that measured the annual increment in clinical severity for individual patients. The only data required to calculate ASIS is the total severity score and the precise age of the patient when the score was ascertained. It is therefore straightforward to calculate on both a retrospective and prospective basis. As long as the clinical scoring system is consistently applied over time to a given patient it also does not matter whether it is based on the original NPC severity score published by Iturriaga and colleagues [16] or the modified clinical severity score devised by Porter and colleagues at the NIH [5] or indeed any other scoring system used locally for patient monitoring.

One important finding in this study was that combining 2–3 subdomain scores correlated well with total severity scores (with and without inclusion of those selected components in the total severity score calculation), suggesting that clinical scoring can be greatly simplified to meet pragmatic constraints in “real life” clinical settings (Additional file 1: Table S1, Additional file 2: Table S2 and Additional file 3: Table S3). It is conceivable that precise single patient sub-score analyses could reduce some of the measurement error associated with the composite score. For example, seizures and cognition, being more binary components, can be confounding in their effects on scores when these domains are included in the total severity score or combined subdomain analysis. Even single sub-scores substituted quite well for total severity scores (Fig. 2 and Additional file 1: Table S1). Based on these findings it may be possible in the future to have regular testing of one or two domains in a home setting thus providing more data points between scheduled clinic visits that often involve travel and disruption of normal daily routine for patients.

These data also provide insights into the pathogenic process as they indicate that the various neurological domains being monitored decline with comparable rates and progress in parallel. Seizures and memory are outliers in this regard. Either other factors contribute to disease progression in these two domains or, more likely, it is the binary nature of seizures (and the effect of seizure medications on seizure scores) and lack of robust ascertainment of declining memory using existing tests that is responsible for their outlier status.

In our previous study, we observed relative stability of ASIS in a very small number of individual patients for whom we had sufficient longitudinal data (10). However, we lacked statistical power due to a limited period of time over which patients were longitudinally monitored. In this study, we therefore collected further longitudinal data points (repeat measures) on a subset of the patients from the original study [10]. The striking finding was that most patients did indeed have relatively stable ASIS scores over time, which suggests that irrespective of their actual rate of progression an individuals’ rate of progression remained fairly constant over time. We do not have sufficient longitudinal data in this study to determine stability over the lifetime of patients, but that could readily be collected both retrospectively and prospectively to determine which subset of patients show changes in rates of clinical progression over time. As might be anticipated, patients that had progressed to develop seizures were more variable, as they represent a more severe and advanced phase of the disease, but many patients with seizures still maintained ASIS score stability (Fig. 3b). The onset of seizures signals a more aggressive phase of the disease [5] [6], but as more treatments are introduced it would be anticipated that the pre-seizure stable ASIS group will expand within the NPC community (due to slowing of disease progression in response to combination therapy). One group underrepresented in this study are adult-onset patients and we plan more in-depth studies in this subgroup of patients in the future.

The main aim of the current study was to compare the potential use of ASIS as a stratification tool for clinical trial recruitment, relative to standard inclusion criteria based on age/neurological signs. To make this more relevant to real-life experience we compared the inclusion criteria used in one historic study in NPC (pivotal miglustat trial, [3]) and two clinical trials in progress at the time of this study (arimoclomol and intra-thecal 2-hydroxypropyl-β-cyclodextrin). All three studies rely on patient age as a primary inclusion criterion, coupled with the need for neurological signs to exclude pre-symptomatic patients. The inclusion criteria for the three trials are summarised in Table 4. It is interesting to note that the neurological inclusion criteria for the three trials differ and are not comparable in their requirements (NCT02612129, NCT02534844). By definition ASIS scores report on neurological signs so the need to specify a particular clinical domain (specific neurological system affected) is not required, greatly simplifying and harmonising the inclusion process. Some specific sub-scores relate more closely to quality of life (QoL). There was consensus between physicians and patient family members who identified the five most important domains as ambulation, swallowing, speech, cognition and fine motor skills. When combined, these five domains correlated well with total severity, whether these components contributed to total severity score or not (correlation coefficients of 0.928 and 0.707 respectively), suggesting they may be the most relevant domains to analyse in clinical trials with direct QoL relevance.

When the three age-based inclusion criteria were compared **(**Table 5**)** the > 12 and 2–18 age inclusion criteria resulted in 76.3% and 57.9% of the cohort being eligible for the trial respectively, whereas 73.7% were eligible with inclusion criteria of 4–21 years. What is key to note is that all three age-based inclusion criteria included patients spanning all clinical severity groups (Fig. 1a-f). We then compared these age inclusion criteria with inclusion based solely on ASIS scores. ASIS resulted in 34–66% eligibility depending on the stringency of the ASIS score thresholds applied. For example, ASIS scores between 0.5–2 resulted in 63% of patients being eligible for inclusion. Applying 0.5–2.5 brings this to 66%. Taken together, applying ASIS as a recruitment tool does result in fewer eligible patients than conventional age/clinical score criteria, but achieves greater clinical homogeneity.

Current clinical trial design for rare diseases is based on placebo-controlled studies as this has proved to be most reliable for common disorders. However, this is challenging in rare diseases due to small patient numbers and considerable clinical heterogeneity [17]. Using patients as their own controls has not yet been incorporated into the design of most rare disease trials, despite its potential merit. The results described in this study suggest that other study designs could be considered. For diseases such as NPC, where patient-held registries have recently been created (International Niemann-Pick Disease Alliance), having a strong body of pre-trial longitudinal data on individual patients will become a realistic prospect for many patients.

Another issue in trial design relates to patient numbers needed for these studies. The miglustat study in NPC was based on pragmatic considerations and no power calculation was used [3]. Power calculations are required by regulators but are of questionable value in rare diseases, as it requires a prediction of effect size to be made based on minimal/no data. If the effect size is large enough, efficacy can be demonstrated with minimal group sizes (e.g. efficacy was demonstrated based on spleen and liver volume reductions in trials of enzyme replacement therapy (ERT) in a non-comparative study of 12 Gaucher disease patients [18]). Indeed, a single patient would have been a good predictor in this case, as no known placebo effect could be ascribed to the dramatic impact of ERT on reducing organomegaly. For trials of disease modifiers with less dramatic effects, stratifying patients and combining analysis of pre- and post-treatment for each patient individually will add greater power, and there will therefore be less chance of missing disease-modifying effects of experimental therapies.

The development of more appropriate patient stratification techniques for recruitment is key to ensure interpretable clinical trial outcomes in the immediate future in rare disease trials. Based on our findings in this report, ASIS may greatly facilitate this process by providing an evidence-based stratification/recruitment tool that is easy to calculate and apply in any clinical setting. It would also allow patient stratification in ongoing trials by allowing the identification of patients at the extremes of the clinical spectrum within the trial cohort, so the data could be analysed with and without their data in a rational, evidence-based and pre-defined and justified, way.

In addition to playing a role in patient stratification, ASIS may also have prognostic value as it should be possible to determine an individual patient’s ASIS and predict probable severity score out into the future. This knowledge would help families plan for the degree and nature of future disability. When we investigated this in our study cohort we found that if we took an average ASIS score based on repeat measurements it compared well with actual measured severity scores over the time period of actual clinical measurements (in this case up to six years). Using a single ASIS determination, as opposed to average ASIS, was less accurate but still had prognostic value. These findings suggest that clinicians, families and patients can also be better informed about clinical trial entry decisions, based on more accurate cost: benefit analysis. For example, if an individual patient is predicted to have a mild clinical course over many years they may opt for less invasive trials than patients with a much more rapidly progressing form of the disease.

It will also be of interest to see if rates of disease progression in other rare diseases, including other lysosomal diseases, are as stable over a number of years as they are in NPC. If that proves to be the case, ASIS may have broader applicability for stratification, recruitment and as a potentially prognostic tool. Finally, we demonstrate the use of ASIS to quantify changes in disease progression in NPC patients associated with treatment with the experimental drug ADLL (Fig. 8). The data gathered over repeat measures over a relatively short time frame showed a significant reduction of approximately 10% in the annual rate of disease progression. This observational study cohort is being followed to generate further longitudinal data to see if this effect is sustained. A placebo-controlled trial is needed to determine efficacy in a rigorous clinical trial setting. ASIS therefore may also be a useful tool for quantifying the effects of therapies and combination therapies in the future, in both observational and pivotal clinical trial settings.

## Conclusion

We have demonstrated that measuring rate of disease progression based on ASIS provides a potentially useful tool for stratifying patents for clinical trials, demonstrating response to therapy and as a prognostic indicator. In addition, we provide evidence that a simplified clinical scoring system based on QoL-related clinical sub-domains is robust and compares well to the full scale that is difficult to ascertain in standard clinical settings. Therefore, taken together these findings will catalyse improvements in clinical trial design/recruitment, act as indicators of prognosis and simplify routine clinical assessments.

## Additional files

Additional file 1: **Table S1.** Spearman’s correlations between the 8 possible subdomains and the total severity score calculated, including or excluding the subdomains in question. (DOCX 15 kb)

Additional file 2: **Table S2.** Spearman’s correlations between the 28 possible pairs of subdomains and the total severity score calculated including or excluding them. (DOCX 16 kb)

Additional file 3: **Table S3.** Spearman’s correlations between the 56 possible triads of subdomains and the total severity score calculated including or excluding the subdomains in question. (DOCX 18 kb)

## Abbreviations

ABR: Auditory brain stem response
ADLL: Acetyl-DL-leucine
ASIS: Annual severity increment score
IQR: Intra-quartile range
MAD: Maximum absolute deviation
NPC: Niemann-Pick disease type C
NPC-CSS: Niemann-Pick disease type C clinical severity score
