# Supplementary material for: Annual severity increment score as a tool for stratifying patients with Niemann-Pick disease type C and for recruitment to clinical trials
Source: Orphanet J Rare Dis. 2018 Aug 16;13:143. doi: 10.1186/s13023-018-0880-9 (PMC6097294; doi:10.1186/s13023-018-0880-9)
Supplement: Supplementary file 1 — Table S1. Spearman’s correlations between the 8 possible subdomains and the total severity score calculated, including or excluding the subdomains in question. (DOCX 15 kb) [file 13023_2018_880_MOESM1_ESM.docx]

|  | Spearman’s correlation coefficients | |
| --- | --- | --- |
| Subdomain (*m*=1) | Including subdomain  in total severity score | Excluding subdomain  in total severity score |
| Eye movement  Ambulation  Speech  Swallow  Fine motor skills  Cognition  Seizures  Memory | 0.534  0.864  0.822  0.728  0.898  0.832  0.600  0.748 | 0.477  0.818  0.792  0.640  0.865  0.811  0.449  0.705 |

**Table S1**: Spearman’s correlations between the 8 possible subdomains

and the total severity score calculated, including or excluding the subdomains in question.
