# Supplementary material for: Annual severity increment score as a tool for stratifying patients with Niemann-Pick disease type C and for recruitment to clinical trials
Source: Orphanet J Rare Dis. 2018 Aug 16;13:143. doi: 10.1186/s13023-018-0880-9 (PMC6097294; doi:10.1186/s13023-018-0880-9)
Supplement: Supplementary file 2 — Table S2. Spearman’s correlations between the 28 possible pairs of subdomains and the total severity score calculated including or excluding them. (DOCX 16 kb) [file 13023_2018_880_MOESM2_ESM.docx]

|  | Spearman’s correlation coefficients | |
| --- | --- | --- |
| Subdomain combination (*m*=2) | Including pair in total severity score | Excluding pair in total severity score |
| Eye movement & Ambulation  Eye movement & Speech  Eye movement & Swallow  Eye movement & Fine motor skills  Eye movement & Cognition  Eye movement & Seizures  Eye movement & Memory  Ambulation & Speech  Ambulation & Swallow  Ambulation & Fine motor skills  Ambulation & Cognition  Ambulation & Seizures  Ambulation & Memory  Speech & Swallow  Speech & Fine motor skills  Speech & Cognition  Speech & Seizures  Speech & Memory  Swallow & Fine motor skills  Swallow & Cognition  Swallow & Seizures  Swallow & Memory  Fine motor skills & Cognition  Fine motor skills & Seizures  Fine motor skills & Memory  Cognition & Seizures  Cognition & Memory  Seizures & Memory | 0.855  0.789  0.778  0.869  0.710  0.835  0.851  0.926  0.880  0.880  0.895  0.917  0.887  0.859  0.933  0.883  0.909  0.930  0.896  0.880  0.856  0.908  0.911  0.925  0.922  0.864  0.922  0.873 | 0.798  0.702  0.669  0.800  0.668  0.764  0.814  0.874  0.784  0.814  0.845  0.834  0.839  0.733  0.874  0.853  0.835  0.903  0.793  0.804  0.735  0.849  0.867  0.839  0.879  0.779  0.900  0.788 |

**Table S2**: Spearman’s correlations between the 28 possible pairs of

subdomains and the total severity score calculated including or excluding them.
