# Supplementary material for: Annual severity increment score as a tool for stratifying patients with Niemann-Pick disease type C and for recruitment to clinical trials
Source: Orphanet J Rare Dis. 2018 Aug 16;13:143. doi: 10.1186/s13023-018-0880-9 (PMC6097294; doi:10.1186/s13023-018-0880-9)
Supplement: Supplementary file 3 — Table S3. Spearman’s correlations between the 56 possible triads of subdomains and the total severity score calculated including or excluding the subdomains in question. (DOCX 18 kb) [file 13023_2018_880_MOESM3_ESM.docx]

|  | Spearman’s correlation coefficient | |
| --- | --- | --- |
| Subdomain combination (*m* = 3) | Including  triad in total  severity score | Excluding  triad in total  severity score |
| Eye movement & Ambulation & Speech  Eye movement & Ambulation & Swallow  Eye movement & Ambulation & Fine motor skills  Eye movement & Ambulation & Cognition  Eye movement & Ambulation & Seizures  Eye movement & Ambulation & Memory  Eye movement & Speech & Swallow  Eye movement & Speech & Fine motor skills  Eye movement & Speech & Cognition  Eye movement & Speech & Seizures  Eye movement & Speech & Memory  Eye movement & Swallow & Fine motor skills  Eye movement & Swallow & Cognition  Eye movement & Swallow & Seizures  Eye movement & Swallow & Memory  Eye movement & Fine motor skills & Cognition  Eye movement & Fine motor skills & Seizures  Eye movement & Fine motor skills & Memory  Eye movement & Cognition & Seizures  Eye movement & Cognition & Memory  Eye movement & Seizures & Memory  Ambulation & Speech & Swallow  Ambulation & Speech & Fine motor skills  Ambulation & Speech & Cognition  Ambulation & Speech & Seizures  Ambulation & Speech & Memory  Ambulation & Swallow & Fine motor skills  Ambulation & Swallow & Cognition  Ambulation & Swallow & Seizures  Ambulation & Swallow & Memory  Ambulation & Fine motor skills & Cognition  Ambulation & Fine motor skills & Seizures  Ambulation & Fine motor skills & Memory  Ambulation & Cognition & Seizures  Ambulation & Cognition & Memory  Ambulation & Seizures & Memory  Speech & Swallow & Fine motor skills  Speech & Swallow & Cognition  Speech & Swallow & Seizures  Speech & Swallow & Memory  Speech & Fine motor skills & Cognition  Speech & Fine motor skills & Seizures  Speech & Fine motor skills & Memory  Speech & Cognition & Seizures  Speech & Cognition & Memory  Speech & Seizures & Memory  Swallow & Fine motor skills & Cognition  Swallow & Fine motor skills & Seizures  Swallow & Fine motor skills & Memory  Swallow & Cognition & Seizures  Swallow & Cognition & Memory  Swallow & Seizures & Memory  Fine motor skills & Cognition & Seizures  Fine motor skills & Cognition & Memory  Fine motor skills & Seizures & Memory  Cognition & Seizures & Memory | 0.891  0.868  0.876  0.879  0.921  0.867  0.839  0.911  0.842  0.915  0.887  0.880  0.839  0.902  0.883  0.902  0.929  0.899  0.869  0.887  0.907  0.928  0.923  0.927  0.956  0.950  0.893  0.907  0.948  0.913  0.900  0.940  0.895  0.917  0.890  0.932  0.918  0.912  0.925  0.926  0.944  0.941  0.941  0.922  0.935  0.940  0.924  0.939  0.923  0.920  0.929  0.939  0.931  0.930  0.931  0.903 | 0.795  0.743  0.770  0.812  0.835  0.804  0.698  0.816  0.763  0.836  0.825  0.760  0.737  0.793  0.804  0.833  0.840  0.828  0.790  0.841  0.836  0.809  0.837  0.863  0.891  0.901  0.756  0.807  0.856  0.817  0.821  0.814  0.799  0.826  0.825  0.847  0.790  0.805  0.815  0.840  0.883  0.847  0.884  0.851  0.903  0.867  0.829  0.830  0.836  0.831  0.869  0.867  0.837  0.876  0.832  0.815 |

**Table S3:** Spearman’s correlations between the 56 possible triads of subdomains and

the total severity score calculated including or excluding the subdomains in question.
